# YOLO-AFL: A Novel Lightweight Algorithm for Real-Time Safety Helmet Detection in Factory Workshops

**DOI:** 10.3390/s26103237

**Published:** 2026-05-20

**Authors:** Hao Wang, Xianying Feng, Peigang Li, Anning Wang, Ming Yao

**Affiliations:** 1 School of Mechanical Engineering, Shandong University, Jinan 250061, China; 2 Key Laboratory of High Efficiency and Clean Mechanical Manufacture of Ministry of Education, Shandong University, Jinan 250061, China

**Keywords:** YOLO, target detection, lightweight model, safety helmet

## Abstract

In factory workshops, wearing safety helmets is vital for worker safety. However, current deep learning-based detection methods are often hindered by large model parameters and high computational demands, limiting their deployment in resource-constrained settings. This article introduces YOLO-AFL, a novel lightweight model designed to solve these problems. The algorithm introduces several key optimizations to improve performance without increasing computational load. Firstly, the K-Means++ algorithm is applied during the anchor box preprocessing stage, along with a new distance metric (1 − AIoU), which enhances anchor box size estimation and boosts performance without additional overhead. Secondly, by introducing a lightweight PConv operation into the C3 module, the complexity of the model is significantly reduced. Finally, a dual attention network (LDA-GC) is designed to compensate for any accuracy loss caused by the model’s simplifications. Experimental results on a custom dataset show that the proposed algorithm achieves an mAP50 of 94.1%. Compared to the baseline model, it reduces the number of parameters by 19.1% and decreases computational complexity by 16.9%, demonstrating its superior performance and efficiency in safety helmet wearing detection.

## 1. Introduction

The factory workshop is the core area of manufacturing and personnel activities, and workshop safety plays a pivotal role in safeguarding worker safety and ensuring the stable operation of enterprises. Although the overall trend of occupational accidents in China has declined in recent years, it still poses a serious threat to safety production in the workshop [1]. Further investigation and analysis have revealed that human, environmental, and equipment factors were the main causes of these accidents. Among them, human factors account for the highest proportion and are especially prominent in complex workshop environments involving hazardous machinery, which is largely due to poor supervision or abnormal behavior [2].

Therefore, it is important to strengthen workshop safety management, and accurate detection of whether employees are wearing helmets can significantly enhance workplace safety [3]. However, the traditional manual supervision method is often time-consuming and laborious, and it is difficult to realize accuracy and real-time safety management. Recent advancements in computer vision technology have facilitated the development of automated helmet detection systems using video surveillance, offering a more efficient solution to mitigate workplace accidents through continuous, real-time monitoring [4].

In traditional research, detection methods often rely on manually engineered features, such as Histogram of Oriented Gradients (HOG), Scale-Invariant Feature Transform (SIFT), and Local Binary Patterns (LBP) [5]. However, these manual feature-based approaches struggle to handle complex and dynamic environments due to challenges posed by factors such as object occlusion, scale variations, and lighting changes. Consequently, with the advancement of deep learning, the powerful feature extraction capabilities of neural networks have introduced a promising approach for safety helmet wearing detection tasks [6].

Due to the complex backgrounds and the presence of numerous interferences in most factory workshops, the accuracy of target detection algorithms is often compromised. To address these issues, previous researchers have primarily focused on optimizing the algorithms. For instance, Chen et al. [7] incorporated Retinex image enhancement technology into the Faster R-CNN framework, effectively reducing missed detections and false positives in helmet-wearing detection. Later, Jin et al. [8] enhanced the Faster R-CNN feature extraction network through feature map fusion, using the improved features for prediction, which significantly boosted model detection accuracy. While these two-stage algorithms demonstrate strong performance, they typically exhibit high model complexity, making them difficult to meet real-time processing requirements.

With the introduction of single-stage object detection algorithms, such as the SSD and YOLO series, several advancements have been made in helmet detection. Li et al. [9] integrated the ShuffleNetv2 feature extraction mechanism within the YOLO framework and combined it with the Efficient Channel Attention (ECA) mechanism, resulting in faster helmet detection. Shen et al. [10] proposed a facial bounding box regression model, which utilizes the DSFD algorithm based on SSD architecture to detect faces to obtain the target location and performs helmet detection through DenseNet network, thus achieving high detection accuracy. Wang et al. [11] developed the YOLO-M helmet detection algorithm, which improves the YOLOv5s backbone by incorporating MobileNetv3 for feature extraction and introducing residual connectivity and the BiCAM attention module, enhancing the detection of small targets. Wu et al. [12] proposed a novel attribute knowledge modeling network based on the Transformer architecture, utilizing a self-attention mechanism to establish relationships between semantic attributes and image features, improving the robustness of worker detection despite appearance variations.

Although recent YOLO-based and lightweight detection models have achieved strong performance, they still face notable limitations in real factory workshop environments. Many approaches rely on large model sizes and high computational costs, which hinder deployment on resource-constrained hardware [13]. Beyond model complexity, several technical issues further affect practical performance. Anchor box generation in most methods depends on Euclidean distance or IoU-based clustering, which is insensitive to aspect ratio variations and leads to inaccurate localization of elongated helmet targets. Moreover, lightweight designs often rely on depth wise or group convolutions to reduce FLOPs, but this can increase memory access overhead and degrade feature representation, resulting in unstable detection under occlusion and dense scenes. In addition, conventional attention mechanisms improve accuracy at the cost of extra computation, conflicting with real-time industrial requirements.

To address these challenges and achieve a better balance between detection accuracy and model complexity, this study proposes targeted improvements to the YOLOv5 (7.0) framework based on both practical deployment constraints and dataset characteristics. We introduce YOLO-AFL, where AFL denotes AIoU, FasterC3, and LDA-GC. By jointly optimizing anchor box clustering, network structure, and feature representation, YOLO-AFL enables fast and efficient safety helmet detection while maintaining high accuracy in factory workshop scenarios.

Although several techniques used in YOLO-AFL are related to existing lightweight convolution and attention mechanisms, this study focuses on their task-oriented adaptation and coordinated design for safety helmet detection in factory workshops. The proposed framework is designed around three requirements of this task: accurate anchor matching for helmet and head targets with varied aspect ratios, reduced computation for real-time deployment, and feature compensation after lightweight network modification. Therefore, the contribution of YOLO-AFL lies not in the independent use of a single module, but in the coordinated optimization of anchor generation, neck structure, and feature representation within a unified YOLOv5-based framework. The main contributions of this paper are summarized as follows:

(1) An optimized anchor box clustering approach is proposed, using K-Means++ algorithm combined with a novel distance metric (1 − AIoU) to improve anchor box size estimation, enhancing localization precision and reducing misalignment without added computational overhead.

(2) A lightweight PConv operation is introduced into the neck network, resulting in the development of a novel FasterC3 module. This modification reduces the number of parameters, while also accelerating the detection speed and decreasing the dependency on high-performance hardware.

(3) To address accuracy losses from network simplification, a new lightweight dual attention network (LDA-GC) is incorporated into the neck structure. LDA-GC enhances feature focus, improving detection performance while keeping computational costs low compared to traditional attention mechanisms.

The remainder of this paper is organized as follows. Section 2 reviews object detection evolution, the YOLOv5 architecture, and attention mechanisms; Section 3 details the YOLO-AFL framework, focusing on anchor optimization, the FasterC3 module, and the LDA-GC network; Section 4 describes the dataset and evaluates performance through ablation and comparative experiments; Section 5 concludes the study and outlines future research directions.

## 2. Related Work

### 2.1. Object Detection

Target detection focuses on identifying the class of objects within images or videos and marking their locations accurately. The introduction of AlexNet [14] in 2012 marked a significant turning point, bringing target detection into the era of deep learning. Following this, two-stage detection algorithms, such as R-CNN [15] and Fast R-CNN [16], demonstrated strong performance by first generating candidate regions and then applying convolutional neural networks (CNNs) to extract features and classify objects. However, the need for separate training in each stage results in slower speeds and higher computational costs.

In contrast, single-stage detection algorithms streamline the process. They simultaneously classify and localize objects in a single pass, enhancing real-time performance and simplifying model design. As a result, algorithms like SSD and the YOLO (You Only Look Once) series have gained popularity for their efficiency and speed, making them a key focus in modern research.

In 2016, Redmon et al. [17] introduced the YOLOv1 algorithm, marking the advent of single-stage object detection. YOLOv1 performs end-to-end detection by dividing an image into grids and using a single neural network to predict bounding boxes and class probabilities simultaneously. Following this, Liu et al. [18] developed the SSD algorithm, which enhanced detection accuracy by leveraging feature information from different layers. Next year, Redmon and colleagues released YOLOv2 [19] and YOLOv3 [20], which introduced innovations like multi-scale fusion and anchor boxes, significantly improving the detection of smaller objects. Since then, the YOLO series has undergone continuous iterations, addressing limitations of earlier versions, such as YOLOv4 [21] and YOLOX [22]. In 2020, Ultralytics made significant advancements by building upon the YOLOv4 architecture. They optimized the network structure, which led to the development of YOLOv5.YOLOv5 has since gained widespread recognition for its enhanced performance and has been broadly adopted in the field of object detection.

### 2.2. YOLOv5

YOLOv5 (7.0) was released in November 2022, offering five different network architectures: n, s, m, l, and x. These models differ in network width and depth, offering flexibility for various performance and resource needs, while maintaining a consistent overall framework. YOLOv5 consists of three main components: the backbone, the neck, and the detection head. The backbone is responsible for extracting essential features from input images, while the neck connects the backbone to the detection head and helps in feature aggregation. The design of this network draws inspiration from FPN [23] and PANet [24]. By integrating pyramid networks, it enhances the interaction of information across different layers effectively. Finally, the detection head is tasked with generating the final object detections by predicting the class and location of each object. This design ensures efficiency and precision in a variety of real-world scenarios.

YOLOv5 (7.0) integrates a wide range of techniques to achieve an optimal balance between detection speed and accuracy. In the backbone network, an innovative SPPF structure is introduced, which significantly improves feature extraction efficiency compared to the original SPP [25]. YOLOv5 (7.0) also incorporates a new C3 module, enhancing the network’s feature representation capabilities. In order to more flexibly adapt to targets of different sizes, YOLOv5 introduces an adaptive anchor box mechanism. To address image deformation issues when resizing input images, YOLOv5 employs a gray padding strategy. This method ensures uniform image dimensions while maintaining the original aspect ratio. Additionally, for the loss function, YOLOv5 (7.0) utilizes CIoU (Complete-IoU) [26] for bounding box regression. Unlike traditional IoU metrics, CIoU not only considers the overlap between boxes but also factors in the distance between center points and the aspect ratio, leading to more stable and accurate bounding box predictions.

### 2.3. Attention Mechanism

In computer vision, the attention mechanism plays a crucial role in processing images and video data. It simulates how humans allocate attention, allowing models to automatically highlight regions of interest and extract more relevant features. In attention mechanisms, the output of each neuron not only depends on the outputs of all neurons in the previous layer but also assigns different weights to different parts of the input data. This enables the model to emphasize key information, improving both accuracy and efficiency. In general, attention mechanisms can be divided into three types according to the principles [27].

**Figure 1:**
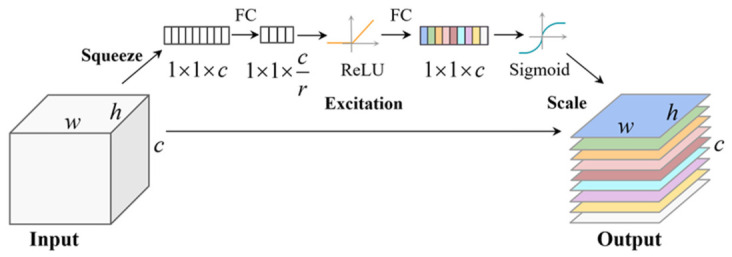
The Squeeze-and-Excitation (SE) attention module.

The spatial attention mechanism allows models to selectively focus on specific objects of interest while suppressing irrelevant background information. By learning and adjusting the weights at each position in the feature map, it enables the model to capture the most relevant spatial features for the task, thus improving the overall detection accuracy. The channel attention mechanism focuses on the relationships and importance among different feature channels in the input data, exemplified by the Squeeze-and-Excitation [28] attention module depicted in Figure 1. It dynamically enhances or suppresses certain channels based on their relevance, allowing the model to improve its feature representation.

However, spatial attention alone does not account for the relationships between channels, while channel attention treats all spatial locations equally. To address these limitations, mixed attention mechanisms like CBAM (Convolutional Block Attention Module) [29] combine both spatial and channel attention. By doing so, CBAM can effectively capture fine-grained information by considering spatial relationships and channel importance separately. This approach allows the model to integrate information more comprehensively.

## 3. Method

### 3.1. YOLO-AFL Model

**Figure 2:**
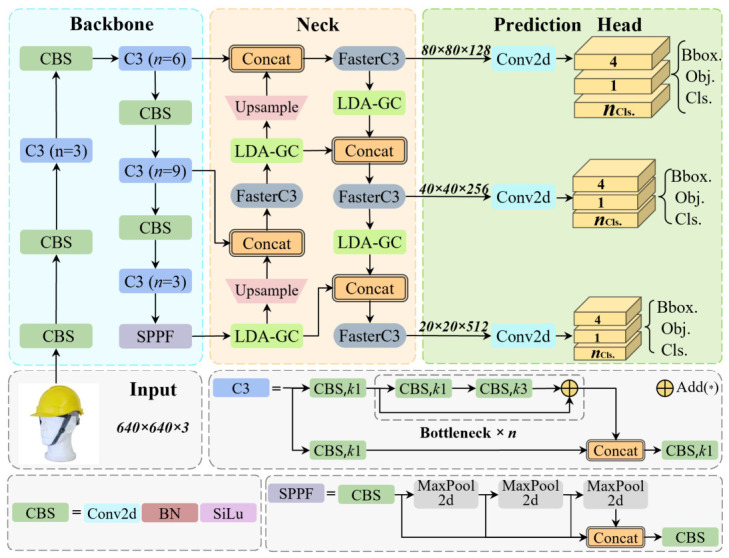
The structure of the improved model YOLO-AFL.

To develop a helmet detection model suitable for complex and resource-constrained workshop environments, we propose YOLO-AFL, a lightweight network built upon the YOLOv5 (7.0) architecture, as illustrated in Figure 2. Compared with the original YOLOv5, YOLO-AFL introduces three targeted architectural modifications at different stages of the network to jointly balance detection accuracy and computational efficiency.

Specifically, at the anchor generation stage, we optimize the default clustering strategy by adopting K-Means++ combined with the proposed AIoU distance metric, enabling better alignment between anchor boxes and ground-truth objects and improving localization accuracy. At the neck stage, the standard C3 modules are replaced with the proposed FasterC3 modules, which employ partial convolution to reduce redundant computation and model parameters while preserving essential feature representations. Furthermore, to compensate for potential feature degradation caused by structural simplification, a lightweight dual-attention module, namely LDA-GC, is introduced into the neck to enhance feature discrimination through grouped channel and spatial attention.

These three modifications are deliberately designed to be complementary rather than independent: anchor optimization improves localization, FasterC3 reduces computational complexity, and LDA-GC restores and strengthens feature representation. The overall architecture thus achieves an effective trade-off between accuracy, speed, and lightweight deployment. The following subsections describe each component in detail.

### 3.2. Clustering Algorithm Optimization

In computer vision, an anchor box is a predefined bounding box fitted to real objects within an image. It is frequently used to predict objects of various sizes, making its dimensions critical to model accuracy. In YOLOv5, anchor boxes are often generated using the K-Means clustering algorithm. The algorithm divides the data into k clusters with similar features by minimizing the distance between samples and centroids.

In the K-Means clustering algorithm, the process begins with randomly selecting k samples from the dataset as initial cluster centers. Next, the distance between each remaining sample and the cluster centers is calculated, assigning each sample to the closest cluster. The algorithm iteratively updates the centroid positions by minimizing the SSE (Sum of Squared Errors) between samples and their respective cluster centers. This process continues until the centroids stabilize or the maximum iteration count is reached. The formula for SSE is defined in Equation (1):

(1) $$\mathrm{SSE}\;=\;\sum_{i=1}^{k}\sum_{X\in C_{i}}|d(X,C_{i}){|}^{2}$$

where *X* and *C_i_* represent the samples and their respective cluster centroids, respectively, and *d* denotes the distance between them.

In the above process, randomness in selecting initial cluster centers may cause all centers to cluster within a limited region, potentially reducing algorithm performance. In contrast, K-Means++ optimizes the selection of initial cluster centers, making the algorithm more likely to reach the global optimum [30]. The core principle of K-Means++ is to maximize the distance between initial cluster centers, with the process as follows:

First, a sample point is randomly chosen from the dataset as the initial cluster center. Second, for each remaining point in the dataset, the shortest distance to every selected center is calculated. Third, new centers are selected based on a probability proportional to this distance, favoring points farther from existing centers. Finally, repeat the above process until k clustering centers are selected, after which the K-Means algorithm generates the final clustering results.

Since each new center is chosen based on its distance from previously selected centers, this approach reduces the likelihood of convergence to a local optimum. For large datasets, K-Means++ significantly reduces training time and resource consumption compared to K-Means, thereby improving target detection efficiency and accuracy [31].

Typically, K-Means++ relies on Euclidean distance to measure differences between two samples. However, this approach tends to introduce more errors with larger bounding boxes compared to smaller ones [19]. Since the goal of clustering is to align anchor boxes more closely with actual target boxes, a metric based on the overlap ratio between two boxes can be more effective. For instance, YOLOv2 employs 1−IoU as a clustering distance measure, where a higher IoU indicates greater similarity between boxes. While this method reduces some errors, it still fails to accurately assess similarity when there is an inclusion relationship between boxes.

**Figure 3:**
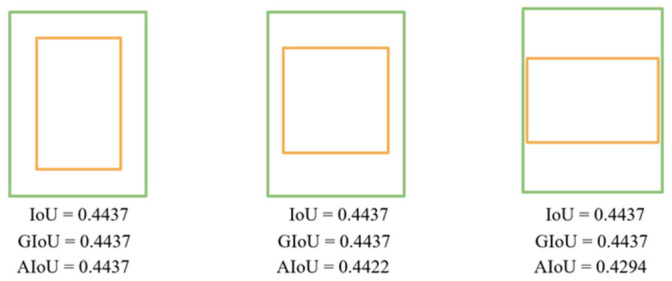
Comparison of results for IoU, GIoU, and AIoU. Orange and green denote anchor box and ground truth box respectively.

Compared with other IoU variants, GIoU (Generalized Intersection over Union) degenerates to IoU when one box is contained within the other, DIoU (Distance-IoU) becomes meaningless since anchor boxes are assumed to share the same center with ground truth boxes during clustering. As illustrated in Figure 3, three orange boxes have identical areas but different aspect ratios. However, each has the same IoU and GIoU with the green box, highlighting the limitations when using IoU and GIoU to distinguish such cases.

Therefore, this study introduces the Aspect-ratio IoU (AIoU), an enhancement of the traditional IoU that incorporates aspect ratio as a significant factor. The calculation formula is given in Equation (2):

(2) AIoU = IoU − αv

where *α* denotes the weighting parameter as defined in Equation (3), and *v* is used to measure the consistency of the aspect ratios between the two boxes as defined in Equation (4):

(3) $$\alpha \;=\;\frac{v}{(1-\mathrm{IoU})\;+\;v}$$

(4) $$\;\;v\;=\frac{4}{\pi^{2}}{\left(\arctan\frac{w^{\mathrm{centroid}}}{h^{\mathrm{centroid}}}\;-\arctan\frac{w^{\mathrm{box}}}{h^{\mathrm{box}}}\right)}^{2}$$

The above expression is inspired by the CIoU loss function, but differs in that AIoU does not incorporate positional information. In AIoU calculations, the centroid and box center are treated as overlapping, which omits the distance term between their centers. During clustering, a higher AIoU between two boxes indicates greater overlap and shape similarity. This design is particularly relevant to safety helmet detection. In workshop images, helmet and head targets often show different scales and aspect ratios due to camera angle, worker posture, partial occlusion, and dense worker distribution. Conventional IoU-based clustering mainly evaluates the overlap area between boxes. As a result, boxes with similar areas but different aspect ratios may obtain similar IoU values, especially when there is an inclusion relationship between boxes. This may reduce the ability of the clustering process to distinguish elongated or shape-varied targets. By introducing aspect ratio consistency into the clustering distance, AIoU helps select anchor boxes that better match the geometric shape of helmet and head targets, thereby reducing anchor-target mismatch during detection.

Table 1 presents a comparison of anchor box sizes and performance metrics obtained using Euclidean distance and 1 − AIoU. Here, BPR (Best Possible Recall) represents the percentage of optimal anchor box matches for each ground truth box that surpass a set threshold (Thr = 2). Fitness measures the suitability of the selected anchor boxes, with higher fitness values indicating a better fit for target boxes in the dataset. We observe that both fitness and BPR increase when 1 − AIoU is used as the distance metric.

### 3.3. FasterC3

**Figure 4:**
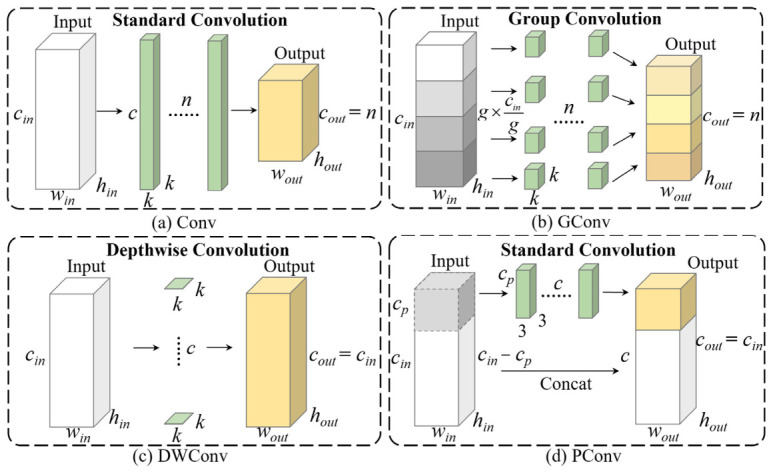
Schematic diagram of various types of convolution.

The goal of constructing lightweight neural networks is to reduce dependency on hardware resources while maintaining high accuracy. Traditional lightweight models often replace standard convolution, as shown in Figure 4a, with GConv or DWConv [32] to reduce computational load, as shown in Figure 4b,c. However, while these methods decrease FLOPs, they increase memory access demands, leading to reduced effective FLOPS. In contrast, FasterNet introduces a novel partial convolution (PConv) that applies standard convolution to only a subset of input channels, preserving information in the remaining channels. This selective convolution approach reduces redundant computations and memory access, achieving greater efficiency [33]. Its structure is shown in Figure 4d.

For an input **I** ∈ *R^h^*^×*w*×*c*^, when using *c* filters of size *k* × *k* and without considering bias and addition operations, the FLOPs for regular Conv, GConv, and PConv are given in Equations (5)–(7):

(5) $$h\;\times \;w\;\times {\;k}^{2}\times \;c^{2}$$

(6) $$h\;\times \;w\;\times \;k^{2}\;\times \;c^{2}\;\times \;\frac{1}{g}$$

(7) $$h\;\times \;w\;\times {\;k}^{2}\;\times \;{c_{p}}^{2}$$

where *g* in Equation (6) represents the number of groups in GConv, and *c_p_* in Equation (7) denotes the number of channels involved in convolution for PConv. When *c_p_* is set to *c*/4, the FLOPs of PConv is only 1/16 of regular Conv, and is also lower than GConv with a typical group count of *g* = 8.

Additionally, the number of memory access for regular Conv, DWConv, and PConv are defined in Equations (8)–(10):

(8) $$h\;\times \;w\;\times \;2c\;+\;k^{2}\;\times \;c^{2}\;\approx \;h\;\times \;w\;\times \;2c$$

(9) $$h\;\times \;w\;\times \;2c^{\prime }+k^{2}\;\times {\;c}^{\prime }\;\approx \;h\;\times \;w\;\times \;2c^{\prime }$$

(10) $$h\;\times \;w\;\times \;2c_{p}+k^{2}\;\times {{\;c}_{p}}^{2}\;\approx \;h\;\times \;w\;\times \;2c_{p}$$

As shown in Equation (9), since DWConv is less effective in feature extraction, the channel count is typically increased to minimize accuracy loss [34], making *c*′ greater than *c*. According to Equation (10), PConv therefore achieves a significant advantage over other convolution operations in terms of the number of memory accesses.

**Figure 5:**
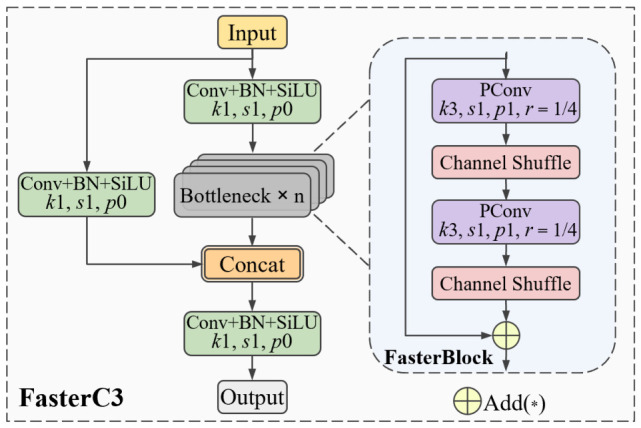
FasterC3 structure.

In YOLOv5, the C3 module is primarily used to increase the receptive field and enhance the model’s feature extraction capability. It consists of multiple stacked Bottleneck blocks, where the convolution operations generate a large number of channels, particularly in deeper layers. These channels often contain highly similar feature maps, resulting in feature redundancy. While this redundancy enables a more comprehensive understanding of the input data, extracting repetitive features also increases unnecessary computational costs [35]. To address this, we replace the CBS module in the Bottleneck with the more lightweight PConv, designing a new feature extraction structure called FasterBlock. We then integrate FasterBlock into the neck network to form the FasterC3 module, as shown in Figure 5.

It can be observed that the FasterBlock module introduces channel shuffle [36] after two partial convolution layers with 3 × 3 kernels. This operation facilitates information exchange between different channels without increasing the model’s size. Subsequent experimental results show that this improvement not only reduces the model’s parameter count and computational burden but also mitigates the significant accuracy loss typically observed in other lightweight models. As a result, it achieves an efficient balance between detection performance and speed.

### 3.4. LDA-GC

To address the potential precision loss from lightweight operations, we have designed a lightweight network called LDA-GC (Lightweight Dual-Attention with Grouping and Channel-shuffle), drawing inspiration from the architectures of CBAM and SA-Net [37]. LDA-GC enhances performance and efficiency by incorporating feature grouping and channel shuffle operations. It also employs parallel channel and spatial attention modules to capture richer semantic information. By focusing limited computational resources on the most significant features, LDA-GC improves the accuracy of safety helmet wearing detection.

**Figure 6:**
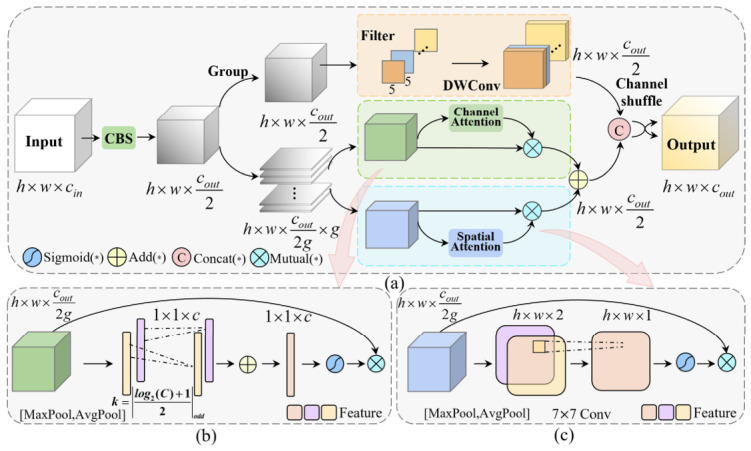
Schematic diagram of LDA-GC structure: (**a**) Overall Structure; (**b**) Channel Attention Module; (**c**) Spatial Attention Module.

As shown in Figure 6a, for an input feature map **F_in_** ∈ *R^h^*^×*w*×*c*^, the CBS module is first used for feature extraction and the channel dimension is adjusted to obtain the input feature map **F** ∈ *R^h^*^×*w*×*c*^*^out^*^/2^ for subsequent modules. Then **F** is enhanced by passing through a DWConv branch and two parallel attention branches. Notably, before entering the attention branches, the features are divided into *g* groups along the channel dimension. This grouping maintains high model performance while minimizing the introduction of additional parameters. In the subsequent parallel branches, the feature map undergoes refinement through channel and spatial attention mechanisms.

These mechanisms apply weighting along the respective dimensions, producing more detailed high-level feature maps, **F_c_** and **F_s_**. Finally, after channel concatenation and shuffling, information from the different branches is fused and exchanged, producing the final output feature map **F_out_** ∈ *R^h^*^×*w*×*c*^*^out^*.

Figure 6b illustrates the process of obtaining the channel attention weighting map **F_c_**. Initially, **F** is processed through global max pooling (GMP) and global average pooling (GAP), generating two feature vectors of size 1 × 1 × *c*, denoted as $F_{GMP}^{c}$ and $F_{GAP}^{c}$. To capture nonlinear cross-channel interactions, a 1D convolution with an adaptive kernel size is used instead of fully connected layers. This avoids the loss of feature extraction efficiency caused by dimensionality reduction [38]. For a feature map with a channel dimension of *c*, the size of the adaptive convolution kernel is defined in Equation (11):

(11) $$\;k\;=\;\psi (c)\;={\left|\frac{\log_{2}(c)}{\gamma }\;+\;\frac{b}{\gamma }\right|}_{\mathrm{odd}}$$

where |*t*|*_odd_* represents the nearest odd number of *t*, *γ* and *b* are commonly set to 2 and 1, respectively.

Ultimately, after Sigmoid activation, the weights are recalibrated to enhance significant spatial features and suppress irrelevant ones. In summary, the process of channel attention weighting is defined in Equation (12):

(12) $$\begin{array}{l} \;F_{c}\;=\;F\cdot \sigma (Conv1D_{k}(MaxPool(F))\;+\;Conv1D_{k}(A\nu gPool(F)))\\ \;\;\;\;\;\;\;=\;F\cdot \sigma (W_{1D}(F_{\mathrm{GMP}}^{c})\;+\;W_{1D}(F_{\mathrm{GAP}}^{c})) \end{array}$$

where *σ* represents the Sigmoid activation function, Conv1D_k_ denotes the nonlinear transformation, and **W_1D_** refers to the weights of the 1D convolution.

For the spatial attention weight map **F_s_**, its generation process is shown in Figure 6c. First, GMP and GAP are applied to learn dependencies in the spatial dimension, producing $F_{GMP}^{s}$ ∈ R*^h^*^×*w*×1^ and $F_{GAP}^{s}$ ∈ R*^h^*^×*w*×1^. Subsequently, they are concatenated into a feature vector with a shape of *h* × *w* × 2, which is then converted to a single channel using a standard 7 × 7 convolution. Finally, the Sigmoid activation function is applied to produce the spatial context descriptor. This descriptor is multiplied with **F** to generate an output with the same shape. The overall process can be described in Equation (13):

(13) $$\begin{array}{l} F_{s}\;=\;F\cdot \sigma (Conv2D_{7\times 7}(Concat(MaxPool(F),A\nu gPool(F))))\\ \;\;\;\;\;=\;F\cdot \sigma (W_{2D}(Concat(F_{\mathrm{GMP}}^{s},F_{\mathrm{GAP}}^{s}))) \end{array}$$

where Conv2D_7×7_ denotes a convolution operation with a kernel size of 7 × 7, Concat signifies the concatenation operation along the channel dimension, and **W_2D_** represents the weights of the convolution operation.

## 4. Experiments

### 4.1. Experimental Environment and Datasets

In this study, all experiments are conducted using RGB images in a PyTorch 1.13.0 environment, with training performed on an RTX 4090D GPU (24 GB) at an input resolution of 640 × 640. To ensure the accuracy and fairness of experimental results, the same platform and training configuration are used for all models during training, validation, and testing phases. Specifically, the training epoch is set to 200, the batch size is 16, the initial learning rate is 0.01, and the momentum parameter for the SGD optimizer is fixed to 0.937.

The specificity of surveillance videos within factory workshops prevents the sharing of data from most actual scenarios. As a result, we chose the publicly available SHWD dataset [39] for this study. This dataset features a vast collection of multi-scene and diverse imagery, which effectively simulates the intricate environmental factors and industrial conditions characteristic of factory workshops.

**Figure 7:**
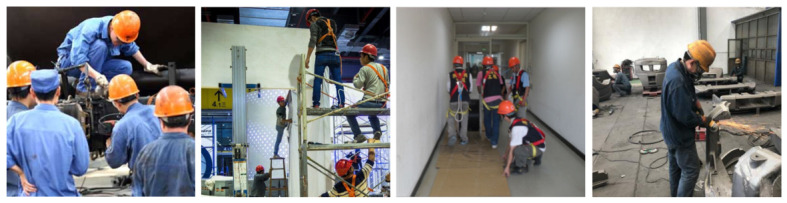
Examples from the SHWD dataset.

The SHWD dataset consists of 7581 images, categorized into workers wearing helmets (“Helmet”) and those without helmets (“Head”), with a focus on head-region detection. Since the original dataset has an unbalanced sample distribution and contains many duplicate images, a strict filtering process was conducted. Low-quality images, duplicate samples, and irrelevant tag types were removed. After filtering, the final refined dataset contained 7200 images. These images were then randomly divided into training, validation, and testing sets in an 8:1:1 ratio while maintaining class balance across each subset. Some sample images from the SHWD dataset are shown in Figure 7.

### 4.2. Experimental Metrics

In deep learning, the confusion matrix is a widely used tool for assessing the performance of classification models. It visually presents the relationship between the model’s predicted outputs and the actual labels in a matrix format, as shown in Table 2.

In order to accurately assess the model’s performance, this study employs several evaluation metrics, including P (Precision), R (Recall), AP (Average Precision), mAP (mean Average Precision), Params (Parameters), and FLOPs (Floating Point Operations).

Precision measures how many of the samples predicted as positive are actually true positives, defined as follows in Equation (14):

(14) $$\mathrm{Precision}\;=\;\frac{\mathrm{TP}}{\mathrm{TP}\;+\;\mathrm{FP}}$$

Recall measures the ability of the model to correctly detect true positive class samples, defined as follows in Equation (15):

(15) $$\mathrm{recall}\;=\;\frac{\mathrm{TP}}{\mathrm{TP}\;+\;\mathrm{FN}}$$

AP is defined as the area under the Precision-Recall curve, and mAP is the average of the AP values calculated for different classes under varying IoU thresholds. The formulae for AP and mAP are given in the following equations in Equations (16) and (17):

(16) $$\mathrm{AP}\;=\;\;\int_{0}^{1}P(r)\mathrm{dr}$$

(17) $$\;\mathrm{mAP}=\frac{1}{N}\sum_{i=1}^{N}AP_{i}$$

where *N* represents the total number of categories in the test set, and *AP_i_* is the AP values for the objects in category *i*.

### 4.3. Ablation Experiment

To validate the detection efficacy of the YOLO-AFL algorithm and the performance improvement contributed by each module, ablation studies were conducted based on the SHWD dataset. The experimental results are presented in Table 3.

As shown in Table 3, the improved anchor clustering method increased mAP50 from 93.3% to 93.8% and mAP50-95 from 61.1% to 61.5%, indicating that the optimized anchor boxes improved the matching between prior boxes and helmet targets. When FasterC3 was used alone, FLOPs decreased from 16.0 G to 13.9 G and Params de-creased from 7.01 M to 6.11 M, while mAP50 decreased slightly to 93.0%. This result shows that FasterC3 effectively reduces model complexity, although partial feature loss may occur due to the lightweight structure. When LDA-GC was introduced alone, mAP50 and mAP50-95 increased to 93.7% and 61.5%, respectively, showing that the attention module improves feature representation.

For the combined settings, Improved Anchor + FasterC3 achieved 93.5% mAP50 and 61.2% mAP50-95, which was higher than FasterC3 alone but lower than Improved Anchor alone. This is because the optimized anchors can partly compensate for the accuracy loss caused by the lightweight FasterC3 structure, but cannot fully recover the reduced feature extraction capacity. Improved Anchor + LDA-GC achieved 94.0% mAP50 and 61.8% mAP50-95, confirming that anchor optimization and attention-based feature enhancement have complementary effects. However, its accuracy was still slightly lower than that of the full YOLO-AFL model, suggesting that FasterC3 also contributes to feature refinement by reducing redundant computation and improving channel information exchange when combined with the other two modules. In addition, FasterC3 + LDA-GC achieved 93.6% mAP50 and 61.3% mAP50-95, which was lower than LDA-GC alone because LDA-GC can only partly recover the feature loss introduced by FasterC3 in the absence of improved anchor matching. Overall, the three modules are not simply additive, since they act on different stages of the detection process. The full YOLO-AFL model achieved the best overall performance, with 94.1% mAP50 and 62.0% mAP50-95, while reducing FLOPs to 13.3 G and Params to 5.67 M. These results show that YOLO-AFL obtains a better balance between detection accuracy and model complexity than the baseline YOLOv5s.

**Figure 8:**
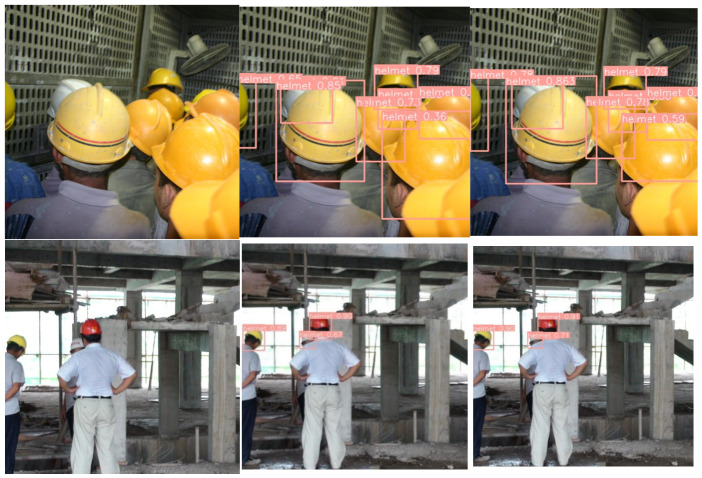

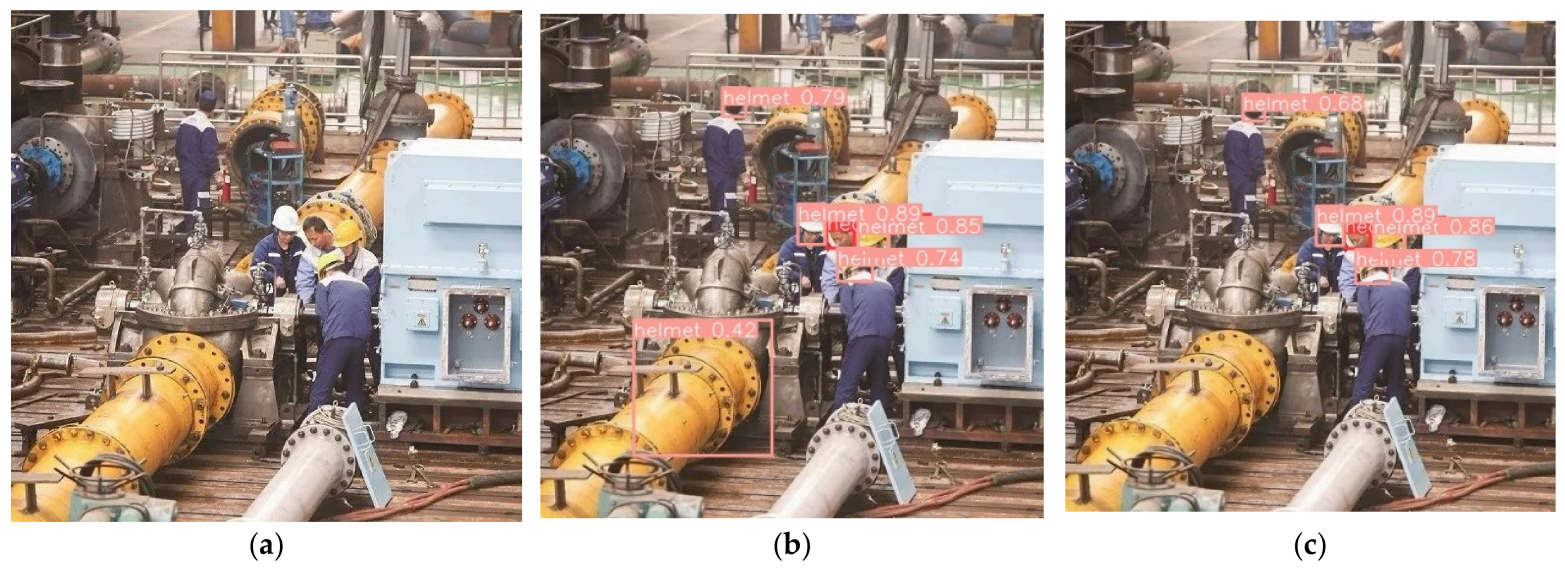
Comparison of detection effect before and after model improvement: (**a**) Original images; (**b**) YOLOv5; (**c**) Ours.

Moreover, to more intuitively perceive the performance advantages of the new model, Figure 8 compares detection results before and after the improvements. The new algorithm demonstrates exceptional performance in enhancing detection accuracy and reducing missed detections, particularly for cluttered backgrounds, small, and occluded objects.

### 4.4. Experiment Results

To thoroughly evaluate the outstanding performance of the YOLO-AFL algorithm in terms of lightweight architecture and computational efficiency, this study also conducted a comparative experiment. Representative lightweight network models from recent years were selected as the Backbone for YOLOv5s, and a comparative analysis was performed using the same test samples. The detailed experimental results are presented in Table 4.

Based on the results in Table 4, YOLO-AFL achieved the best detection accuracy among the compared lightweight models, with 94.1% mAP50 and 62.0% mAP50-95. Compared with the YOLOv5s baseline, YOLO-AFL reduced Params from 7.01 M to 5.67 M and FLOPs from 16.0 G to 13.3 G, while increasing mAP50 from 93.3% to 94.1%. This result shows that the proposed method improves detection accuracy while reducing model complexity. In contrast, several lightweight backbones, such as MobileNetV3 and ShuffleNetV2, reduced FLOPs and Params more clearly but caused a notable decrease in detection accuracy. Therefore, the advantage of YOLO-AFL does not lie in minimizing model size alone, but in maintaining a better balance between accuracy and computational cost.

It should also be noted that the inference time improvement on the RTX 4090D is limited. YOLO-AFL reduced the inference time from 4.0 ms to 3.8 ms compared with YOLOv5s. This is because inference latency on a high-end GPU is affected not only by FLOPs and Params, but also by CUDA kernel launch overhead, memory access, parallel computing efficiency, data preprocessing, and post-processing. The reduction in Params and FLOPs may provide greater benefits on resource-constrained edge devices, where memory access and bandwidth are more limited.

Additionally, to comprehensively evaluate the performance of the improved YOLOv5 network in the workshop employee safety helmet wearing detection task, this study also selected several classic object detection networks from recent years as benchmark models for comparison. The specific experimental results are as follows.

Table 5 compares YOLO-AFL with several representative object detection models in terms of precision, recall, mAP50, FLOPs, parameter count, and inference time. Since different detectors have different model scales and design strategies, the results in Table 5 are mainly used to compare the trade-off among accuracy, computational cost, model size, and inference speed under the same dataset and testing platform.

Among the compared models, YOLOv5 has relatively low computational complexity, with 16.0 GFLOPs and 7.01 M parameters, but its mAP50 is 93.3%, which is lower than that of YOLO-AFL. YOLOv7 achieves a competitive mAP50 of 93.7% and a high recall value, but it requires 105.1 GFLOPs and 37.20 M parameters, which limits its use in resource-constrained deployment scenarios. To further evaluate the proposed method, YOLO-AFL was also compared with more recent YOLO models, including YOLOv10 and YOLO26. YOLOv10 achieved 94.0% mAP50 with 24.4 GFLOPs and 8.04 M parameters, while YOLO26 achieved 94.2% mAP50 with 22.5 GFLOPs and 9.95 M parameters. These models show competitive detection accuracy, but their computational costs and parameter counts are higher than those of YOLO-AFL.

In contrast, YOLO-AFL achieved 94.1% mAP50 with 13.3 GFLOPs and 5.67 M parameters. Although YOLO26 achieved a slightly higher mAP50, YOLO-AFL required fewer parameters and lower computational cost, indicating a better balance between detection accuracy and lightweight deployment. It also achieved an inference time of 3.8 ms, which was faster than YOLOv5, YOLOv8, YOLOv10, and YOLO26 under the same testing conditions. Compared with YOLOv5, YOLO-AFL improved mAP50 by 0.8% while reducing FLOPs by 16.9% and parameters by 19.1%. Although this accuracy improvement is moderate, it is meaningful for continuous safety monitoring in factory workshops, where missed detections may accumulate over a large number of worker appearances. In addition, the reduced model size and computational cost can help lower memory usage and improve deployment potential on resource-constrained devices. Therefore, YOLO-AFL provides a practical balance among detection accuracy, model complexity, and inference efficiency for real-time safety helmet detection.

## 5. Conclusions

In summary, this study integrates various improvements into the YOLOv5 base model, designing a lightweight safety helmet detection algorithm specifically for workshop scenarios. Specifically, the K-Means++ algorithm and the 1 − AIoU distance metric are employed to optimize the selection of initial anchor boxes, enabling the model to locate targets more precisely. The introduction of the FasterC3 module significantly reduces model complexity without overly compromising detection performance. Additionally, LDA-GC enhances the detection accuracy and computational efficiency of the model by suppressing non-critical features and focusing on core information. These improvements result in a model that not only inherits but surpasses the performance of YOLOv5, accomplishing a lightweight architecture while maintaining exceptional detection precision.

Experiments show that the new algorithm reduces parameters by 19.1%, decreases computational complexity by 16.9%, and shortens inference time by 0.2 ms compared to YOLOv5. It also achieves a mean average precision of 94.1% at an IoU threshold of 0.5. Performance evaluation on the same dataset demonstrates that the proposed algorithm offers superior overall performance compared to other neural network models. This highlights its ability to maintain high detection accuracy in resource-constrained environments. Therefore, the proposed algorithm meets the demands for high-accuracy and low-cost safety helmet wearing detection in factory workshop environments.

In future research, we will continue to explore more advanced techniques to further optimize the model structure and improve its real-time performance and accuracy. Furthermore, we will attempt to deploy the model on embedded edge devices such as NVIDIA Jetson to enable immediate on-site processing, thereby further reducing the demand for computational resources.

## Figures and Tables

**Table 1:** Comparison of anchor box metrics: Euclidean vs. 1 − AIoU.

Distance Metric	Clusters	Anchor	Thr	Fitness	BPR
Euclidean distance	k = 9	(9, 10) (12, 14) (16, 18) (23, 25) (33, 38) (51, 56) (79, 93) (132, 156) (276, 304)	2	0.8303	0.998170
1 − AIoU	k = 9	(9, 10) (11, 13) (15, 18) (21, 25) (32, 36) (47, 54) (75, 87) (128, 155) (303, 332)	2	0.8328	0.999516

**Table 2:** Confusion matrix.

Actual Labels	Predicted Outputs	
	Positive	Negative
Positive	True Positive (TP)	False Negative (FN)
Negative	False Positive (FP)	True Negative (TN)

**Table 3:** The ablation experiment results.

Improved Anchor	FasterC3	LDA-GC	mAP50 (%)	mAP50-95 (%)	FLOPs (G)	Params (M)
			93.3	61.1	16.0	7.01
√			93.8	61.5	16.0	7.01
	√		93.0	60.9	13.9	6.11
		√	93.7	61.5	15.2	6.58
√	√		93.5	61.2	13.9	6.11
√		√	94.0	61.8	15.2	6.58
	√	√	93.6	61.3	13.3	5.67
√	√	√	94.1 (↑ 0.8)	62.0 (↑ 0.9)	13.3 (↓ 2.7)	5.67 (↓ 1.34)

Note: “√” indicates that the corresponding module is included. “↑” and “↓” indicate increases and decreases compared with the baseline model YOLOv5s, respectively.

**Table 4:** Performance comparison of lightweight network models.

Models	mAP50 (%)	mAP50-95 (%)	Inference Time (ms)	FLOPs (G)	Params (M)
Baseline	93.3	61.1	4	16.0	7.01
GhostNetV2	90.5	59.9	3.6	12.3	5.69
MobileNetV3 [40]	88.0	54.5	3.4	6.3	3.55
EfficientViT [41]	88.3	57.6	4.8	14.7	5.96
ShuffleNetV2 [42]	87.4	55.8	3.3	8.0	3.80
EfficientNet [43]	91.3	60.2	7.2	14.4	9.11
Ours	94.1	62.0	3.8	13.3	5.67

**Table 5:** Performance comparison of classical target detection algorithms.

Models	Precision (%)	Recall (%)	mAP50 (%)	Inference Time (ms)	FLOPs (G)	Params (M)
YOLOv5	91.8	87.6	93.3	4.0	16.0	7.01
Faster R-CNN	53.5	84.7	79.6	18.5	137.2	41.4
YOLOv3-tiny	90.8	85.6	86.3	4.3	19.0	12.13
YOLOX	88.1	77.4	92.6	5.6	26.8	8.94
YOLOv7 [44]	92.2	90.1	93.7	12.4	105.1	37.20
RT-DETR [45]	91.7	87.4	93.0	10.8	86.2	32.76
YOLOv8	92.3	88.9	93.8	5.2	28.4	11.13
YOLOv10 [46]	92.9	89.3	94.0	4.8	24.4	8.04
YOLO26 [47]	93.0	89.7	94.2	5.0	22.5	9.95
Ours	93.2	89.5	94.1	3.8	13.3	5.67

## Data Availability

Data derived from public domain resources. The Safety Helmet Wearing Dataset (SHWD) used in this study is publicly available at the following URL: https://github.com/njvisionpower/Safety-Helmet-Wearing-Dataset (accessed on 10 March 2025).

## References

[1] Luo X.X., Li X.C., Goh Y.M., Song X.F., Liu Q.L. (2023). Application of Machine Learning Technology for Occupational Accident Severity Prediction in the Case of Construction Collapse Accidents. Saf. Sci..

[2] Zhou F.J., Zhang J., Fu C. (2023). Generation Paths of Major Production Safety Accidents—A Fuzzy-Set Qualitative Comparative Analysis Based on Chinese Data. Front. Public Health.

[3] Lyu Y.K., Yang X.B., Guan A., Wang J.W., Dai L.N. (2024). Construction Personnel Dress Code Detection Based on YOLO Framework. CAAI Trans. Intell. Technol..

[4] Vukicevic A.M., Petrovic M., Milosevic P., Peulic M., Jovanovic K., Novakovic A. (2024). A Systematic Review of Computer Vision-Based Personal Protective Equipment Compliance in Industry Practice: Advancements, Challenges and Future Directions. Artif. Intell. Rev..

[5] Kapse A.S., Shreevamshi, Ravichandra P., Reddy R., Reddy R. (2023). A Survey on Helmet Detection by CNN Algorithm. ITM Web Conf..

[6] Imam M., Baïna K., Tabii Y., Ressami E.M., Adlaoui Y., Boufousse S., Benzakour I., Abdelwahed E.H. (2025). Integrating Real-Time Pose Estimation and PPE Detection with Cutting-Edge Deep Learning for Enhanced Safety and Rescue Operations in the Mining Industry. Neurocomputing.

[7] Chen S.B., Tang W.H., Ji T.Y., Zhu H.L., Ouyang Y., Wang W.B. Detection of Safety Helmet Wearing Based on Improved Faster R-CNN. Proceedings of the 2020 International Joint Conference on Neural Networks (IJCNN).

[8] Jin M., Lu B., Zhang J., Wang Q., Chen X.W., Nie G.N. Video Streaming Helmet Detection Algorithm Based on Feature Map Fusion and Faster Rcnn. Proceedings of the 2021 International Conference on Electronic Information Engineering and Computer Science (EIECS).

[9] Li S.Y., Lv Y.C., Liu X.Y., Li M.F. (2023). Detection of Safety Helmet and Mask Wearing Using Improved YOLOv5s. Sci. Rep..

[10] Shen J., Xiong X., Li Y., He W., Li P., Zheng X.Y. (2021). Detecting Safety Helmet Wearing on Construction Sites with Bounding-Box Regression and Deep Transfer Learning. Comput. Aided Civ. Infrastruct. Eng..

[11] Wang L.L., Zhang X.J., Yang H.L. (2023). Safety Helmet Wearing Detection Model Based on Improved YOLO-M. IEEE Access.

[12] Wu X., Li Y.P., Long J.H., Zhang S., Wan S., Mei S.H. (2023). A Remote-Vision-Based Safety Helmet and Harness Monitoring System Based on Attribute Knowledge Modeling. Remote Sens..

[13] Mittal P. (2024). A Comprehensive Survey of Deep Learning-Based Lightweight Object Detection Models for Edge Devices. Artif. Intell. Rev..

[14] Krizhevsky A., Sutskever I., Hinton G.E. (2017). ImageNet Classification with Deep Convolutional Neural Networks. Commun. ACM.

[15] Girshick R., Donahue J., Darrell T., Malik J. Rich Feature Hierarchies for Accurate Object Detection and Semantic Segmentation. Proceedings of the IEEE Conference on Computer Vision and Pattern Recognition (CVPR).

[16] Ren S.Q., He K.M., Girshick R., Sun J. (2016). Faster R-CNN: Towards Real-Time Object Detection with Region Proposal Networks. IEEE Trans. Pattern Anal. Mach. Intell..

[17] Redmon J., Divvala S., Girshick R., Farhadi A. You Only Look Once: Unified, Real-Time Object Detection. Proceedings of the IEEE Conference on Computer Vision and Pattern Recognition (CVPR).

[18] Liu W., Anguelov D., Erhan D., Szegedy C., Reed S., Fu C.Y., Berg A.C. SSD: Single Shot Multibox Detector. Proceedings of the 14th European Conference on Computer Vision (ECCV).

[19] Redmon J., Farhadi A. YOLO9000: Better, Faster, Stronger. Proceedings of the IEEE Conference on Computer Vision and Pattern Recognition (CVPR).

[20] Redmon J., Farhadi A. (2018). YOLOv3: An Incremental Improvement. arXiv.

[21] Bochkovskiy A., Wang C.Y., Liao H.Y.M. (2020). YOLOv4: Optimal Speed and Accuracy of Object Detection. arXiv.

[22] Ge Z., Liu S.T., Wang F., Li Z.M., Sun J. (2021). YOLOX: Exceeding YOLO Series in 2021. arXiv.

[23] Lin T.Y., Dollár P., Girshick R., He K., Hariharan B., Belongie S. Feature Pyramid Networks for Object Detection. Proceedings of the IEEE Conference on Computer Vision and Pattern Recognition (CVPR).

[24] Liu S., Qi L., Qin H.F., Shi J.P., Jia J.Y. Path Aggregation Network for Instance Segmentation. Proceedings of the IEEE Conference on Computer Vision and Pattern Recognition (CVPR).

[25] He K.M., Zhang X.Y., Ren S.Q., Sun J. (2015). Spatial Pyramid Pooling in Deep Convolutional Networks for Visual Recognition. IEEE Trans. Pattern Anal. Mach. Intell..

[26] Zheng Z.H., Wang P., Liu W., Li J.Z., Ye R.G., Ren D.W. Distance-IOU Loss: Faster and Better Learning for Bounding Box Regression. Proceedings of the AAAI Conference on Artificial Intelligence.

[27] Niu Z.Y., Zhong G.Q., Yu H. (2021). A Review on the Attention Mechanism of Deep Learning. Neurocomputing.

[28] Hu J., Shen L., Sun G. Squeeze-and-Excitation Networks. Proceedings of the IEEE Conference on Computer Vision and Pattern Recognition (CVPR).

[29] Woo S., Park J., Lee J.Y., Kweon I.S. CBAM: Convolutional Block Attention Module. Proceedings of the 15th European Conference on Computer Vision (ECCV).

[30] Arthur D., Vassilvitskii S. K-Means++: The Advantages of Careful Seeding. Proceedings of the Eighteenth Annual ACM-SIAM Symposium on Discrete Algorithms (SODA).

[31] Zhang B., Sun C.F., Fang S.Q., Zhao Y.H., Su S. (2022). Workshop Safety Helmet Wearing Detection Model Based On SCM-YOLO. Sensors.

[32] Howard A.G., Zhu M.L., Chen B., Kalenichenko D., Wang W.J., Weyand T., Andreetto M., Adam H. (2017). MobileNets: Efficient Convolutional Neural Networks for Mobile Vision Applications. arXiv.

[33] Chen J.R., Kao S.H., He H., Zhuo W.P., Wen S., Lee C.H. Run, Don’t Walk: Chasing Higher FLOPS for Faster Neural Networks. Proceedings of the IEEE/CVF Conference on Computer Vision and Pattern Recognition (CVPR).

[34] Sandler M., Howard A., Zhu M.L., Zhmoginov A., Chen L.C. MobileNetV2: Inverted Residuals and Linear Bottlenecks. Proceedings of the IEEE Conference on Computer Vision and Pattern Recognition (CVPR).

[35] Tang Y.H., Han K., Guo J.Y., Xu C., Xu C., Wang Y.H. GhostNetV2: Enhance Cheap Operation with Long-Range Attention. Proceedings of the 36th International Conference on Neural Information Processing Systems (NeurIPS).

[36] Zhang X.Y., Zhou X.Y., Lin M.X., Sun J. ShuffleNet: An Extremely Efficient Convolutional Neural Network for Mobile Devices. Proceedings of the IEEE Conference on Computer Vision and Pattern Recognition (CVPR).

[37] Zhang Q.L., Yang Y.B. SA-Net: Shuffle Attention for Deep Convolutional Neural Networks. Proceedings of the 2021 IEEE International Conference on Acoustics, Speech and Signal Processing (ICASSP).

[38] Wang Q.L., Wu B.G., Zhu P.F., Li P.H., Zuo W.M., Hu Q.H. ECA-Net: Efficient Channel Attention for Deep Convolutional Neural Networks. Proceedings of the IEEE/CVF Conference on Computer Vision and Pattern Recognition (CVPR).

[39] Njvisionpower (2024). Safety-Helmet-Wearing-Dataset (SHWD).

[40] Howard A., Sandler M., Chen B., Wang W.J., Chen L.C., Tan M.X. Searching for MobileNetV3. Proceedings of the IEEE/CVF International Conference on Computer Vision (ICCV).

[41] Liu X.Y., Peng H.W., Zheng N.X., Yang Y.Q., Hu H., Yuan Y.X. EfficientViT: Memory Efficient Vision Transformer with Cascaded Group Attention. Proceedings of the IEEE/CVF Conference on Computer Vision and Pattern Recognition (CVPR).

[42] Ma N.N., Zhang X.Y., Zheng H.T., Sun J. ShuffleNet V2: Practical Guidelines for Efficient CNN Architecture Design. Proceedings of the European Conference on Computer Vision (ECCV).

[43] Tan M.X., Le Q.V. EfficientNet: Rethinking Model Scaling for Convolutional Neural Networks. Proceedings of the International Conference on Machine Learning (ICML).

[44] Wang C.Y., Bochkovskiy A., Liao H.Y.M. YOLOv7: Trainable Bag-of-Freebies Sets New State-of-the-Art for Real-Time Object Detectors. Proceedings of the IEEE/CVF Conference on Computer Vision and Pattern Recognition (CVPR).

[45] Zhao Y., Lv W.Y., Xu S.L., Wei J.M., Wang G.Z., Dang Q.Q. DETRs Beat YOLOs On Real-Time Object Detection. Proceedings of the IEEE/CVF Conference on Computer Vision and Pattern Recognition (CVPR).

[46] Wang A., Chen H., Liu L.H., Chen K., Lin Z.J., Han J.G., Ding G.G. YOLOv10: Real-Time End-to-End Object Detection. Proceedings of the 38th International Conference on Neural Information Processing Systems (NeurIPS).

[47] Sapkota R., Cheppally R.H., Sharda A., Karkee M. (2025). YOLO26: Key architectural enhancements and performance benchmarking for real-time object detection. arXiv.

